# The influencing factors of the participation intention and behavior in organized scientific research of young teachers in modern industrial colleges—based on the theory of planned behavior and normative activation theory

**DOI:** 10.3389/fpsyg.2025.1450240

**Published:** 2025-02-04

**Authors:** Yuanbao Zhang, Junfeng Li, Jinyu Song

**Affiliations:** Changshu Institute of Technology, Suzhou, China

**Keywords:** Modern Industrial College, organized scientific research, participation behavior, theory of planned behavior, normative activation theory, young teachers

## Abstract

Organized scientific research is vital to implementing organized technological innovation in modern industrial colleges. Young teachers’ deep participation determines success. Based on the theory of planned behavior and normative activation, a survey of 309 young teachers from modern industrial colleges participating in organized scientific research found that personal norms, subjective norms, behavioral attitudes, and perceived behavioral control significantly impact their intention to participate. Awareness of consequence, ascribed responsibility, and subjective norms indirectly positively impact their participation intention, and the participation intention and perceived behavioral control positively affect their participation behavior. Policy support has a positive mediating effect between participation intention and behavior. There are group differences in gender, education level, professional title, and subject type in demographic characteristics in five aspects: subjective norms, personal norms, perceived behavioral control, participation intention, and participation behavior. This article proposes the following measures: improving the internal interest coordination mechanism of scientific research organizations to enhance young teachers’ sense of scientific research achievement; reconstructing the organized scientific research evaluation model to enhance young teachers’ teamwork awareness; improving the support system for organized research policies to stimulate young teachers’ self-efficacy; concerning young teachers’ group differences and creating a research environment that tolerates failure.

## Introduction

As the new scientific-technological and industrial revolutions are booming, the breadth and depth of technological innovation have significantly increased, the speed has accelerated, and the accuracy has strengthened dramatically. Various original and fundamental technological breakthroughs increasingly require organized scientific research activities. Chinese universities’ scientific and technological innovation organizations need more systematic layouts and support for primary national strategic needs. To crack the current problems, in 2022, The Ministry of Education issued “Several opinions on strengthening organized scientific research in universities to promote high-level self-reliance and self-improvement, “which point out: “Universities are an important component of the national strategic scientific and technological strength, the main force of basic research, and the source of major scientific and technological breakthroughs… To accelerate the transformation of university research paradigms and organizational models, strengthen organized scientific research, better serve the practical problems and urgent needs faced by national security and economic and social development, and provide strong support for achieving high-level scientific and technological self-reliance, accelerating the construction of world important talent centers and innovation highlands.” Organized scientific research, as an important form of institutional and systematic service for national and regional strategic needs in scientific and technological innovation in universities, revolves around national science and technology’s forefront and significant needs. Strategic layout and standardized design continuously strengthen the degree of organization and coordination of scientific research activities to improve the overall efficiency of national scientific and technological innovation. As an important carrier for implementing organized scientific research in universities, the participation of young teachers in modern industrial colleges is not only related to the smooth implementation of the organized scientific research system but also to the future survival and high-quality development of a university.

Domestic research on organized scientific research mainly focuses on development concepts, organizational models, implementation difficulties, development mechanisms, and promotion strategies. In development concepts, organized scientific research in universities should adhere to mission orientation and focus on solving major national technological and social challenges; it should assume social responsibility and focus on the value pursuit of enhancing human well-being; it should adhere to free exploration and promote the high-quality development of the scientific and technological innovation system (Wu and Shi, 2023) In organizational models, domestic universities mainly adopt main organizational models such as platformization, specialization, institutionalization, groundation, centralization, and school-enterprise collaboration (Chen, 2023). Currently, value fragmentation, institutional fragmentation, technological fragmentation, and subject fragmentation are the main challenges faced by Chinese universities in implementing organized scientific research. Among them, value fragmentation is mainly reflected in the gradual distance between teachers’ scientific research work and academic essence, institutional fragmentation is mainly reflected in the unsystematic supporting policies for scientific research in universities, technological fragmentation is mainly reflected in the data “islands” between different researchers, and subject fragmentation is mainly reflected in the difficulty of integrating the interests and demands of different scientific research subjects (Huang and Xiao, 2023). To address these challenges, it is necessary to continuously strengthen mechanism innovation in resource allocation, research driving force, organizational coordination, research governance, and research evaluation (Bai, 2023). It is also necessary to comprehensively promote the reform of the scientific research system, the construction of scientific research platforms, and the creation of a cultural atmosphere in universities (Shi and Zhou, 2023).

Foreign scholars have conducted in-depth discussions on organized scientific research from two aspects: why and how. Bourdieu (2005) points out that a vital scientific research paradigm is that, under specific spatiotemporal conditions, specific research groups engage in collaborative cooperation by intervening in the “field.” Clark (1998) believes organized scientific research can break down barriers between disciplines, help unleash team power and collective wisdom, and become a driving force for knowledge innovation in modern universities. Rhoten and Parker (2004) further elaborated on this, stating that members of an organized research team have a shared vision and goals, similar research paradigms and disciplinary thinking, shared knowledge systems, and cultural identity, which are more conducive to improving the efficiency of knowledge innovation. Especially with the transformation of knowledge production methods, collaborating with different “academic tribes” to conduct interdisciplinary research has become a meaningful way to solve complex problems in contemporary society, and the basis for conducting multidisciplinary research is an effective organization of scientific research activities (Holly, 2012). Geiger (1990) found in his study on the transfer history of world science centers that the transfer of world science centers ultimately stems from the support and effective organization of countries for scientific research activities such as knowledge production and technological innovation. The book “Science: Endless Frontiers.” confirmed this conclusion. Bush and Holt (2021) found that the federal government effectively organizes major technological innovations that serve national strategic needs, laying a solid foundation for the prosperity of scientific research and economic and social development in the United States and profoundly influencing the global pattern of technological development.

In summary, we can see that researchers in China and abroad mainly focus on constructing an ideal organized scientific research form from the “should be” level in terms of content while emphasizing speculative research and logical reasoning in terms of methods, lacking empirical research on the participants of organized scientific research. Especially for the young scientific research “green pepper” group in modern industrial colleges, are they willing to participate in organized scientific research? What factors affect their participation in organized scientific research, and what are their concerns? What are the collective characteristics of these young teachers? These issues are unavoidable for domestic universities when carrying out organizational research. This study uses young teachers from seventeen “National First Batch of Modern Industry Colleges” in the Yangtze River Delta region of China as research samples based on the theory of planned behavior and normative activation. It attempts to reveal the influencing factors and mechanisms of young teachers’ participation in organized scientific research through empirical research to promote further research by offering valuable insights, as wells as explore ways to further promote the organization and collaboration of scientific research activities in modern industrial colleges, and enhance the overall efficiency of scientific and technological innovation in modern industrial colleges.

## Theoretical model and hypothesis development

### Theory of planned behavior and its variable relationships

The theory of Planned Behavior (TPB) is a social psychology theory proposed by American scholar Ayez based on the Theory of Rational Behavior (TRA). It is an important theoretical basis for explaining and predicting the behavior of rational individuals. This theory suggests that the behavioral decision-making of “rational individuals” is mainly influenced by behavioral intention, mainly by three factors: behavioral attitude, subjective norms, and perceived behavioral control (Allen and Marquart-Pyatt, 2018). Among them, intention reflects the subjective effort level of the behavioral subject toward implementing a specific behavior; behavioral attitude reflects the preference level of the behavioral subject toward implementing a certain behavior; subjective norms reflect the external pressure felt by the behavioral subject toward implementing a specific behavior, and perceived behavioral control reflects the personal judgment of the behavioral subject on the difficulty level of implementing a particular behavior (Elliott et al., 2007). Behavioral attitudes, subjective norms, and perceived behavioral control have a significant positive impact on the entrepreneurial intention of young university researchers (Rosangela et al., 2019). Behavioral intention and perceived behavioral control have a significant positive effect on the participation of enterprises in school-enterprise cooperation behavior (Ran, 2021). It is thus clear that the Theory of Planned Behavior predicts individual behavior and its antecedent influencing factors from the perspective of the psychological structure of rational individuals. Therefore, it is also appropriate to explore the participation of young teachers in organized scientific research in modern industrial colleges. Young teachers in modern industrial colleges face varying degrees of pressure in scientific research assessment, and organized scientific research is an important organizational form to improve the efficiency of scientific research activities and alleviate assessment pressure. When young teachers are satisfied with this corporate form, other teachers can also actively participate, and they have the internal and external conditions to experience; their intention to participate will be strong, and the possibility of taking practical actions will be greater. In addition, another scholar pointed out that situational factors are important external incentives for behavioral subjects to transform their intentions into practical actions (Stern, 2000). For young teachers in modern industrial colleges, policy support may encourage them to obtain more external benefits, thereby stimulating them to transform their intention to participate in organized scientific research into actual participation behavior. Based on this, this study proposes the following hypothesis:

*H1*: Subjective norms have a positive impact on the intention of young teachers to participate in organized scientific research.

*H2*: Behavioral attitudes have a positive impact on the intention of young teachers to participate in organized scientific research.

*H3*: Perceived behavioral control has a positive impact on the intention of young teachers to participate in organized research.

*H4*: Perceived behavioral control has a positive impact on the participation of young teachers in organized scientific research behavior.

*H5*: The participation intention has a positive impact on the organized research behavior of young teachers.

*H6*: Policy support has a positive moderating effect on the intention and behavior of young teachers to participate in organized research.

### Normative activation theory and its variable relationships

Normative Activation Theory is a social psychology theory that predicts and explains individual altruistic behavior based on social obligations and moral norms. This theory suggests that although society encourages altruistic behavior, it does not necessarily mean everyone can obey social domination. Altruistic behavior results from individuals internalizing external norms, mainly influenced by their awareness of consequences (AC) and ascribed responsibility (AR). The stronger the individual’s awareness of consequences and ascribed responsibility is, the greater the possibility of forming certain norms (Schwartz, 1973). Among them, the awareness of consequences reflects an individual’s subjective judgment on the consequences of implementing or not implementing a specific behavior, the ascribed responsibility demonstrates an individual’s belief that they should take corresponding responsibility for the adverse consequences of a particular behavior, and personal norms reflect an individual’s self-expectation and sense of obligation to implement a specific behavior. Present studies indicate that a certain chain relationship between awareness of consequences, ascribed responsibility, personal norms, and behavioral intention; that is, an individual’s behavioral intention forms through the chain effect of “awareness of consequence-ascribed responsibility-personal norms” (Steg and De Groot, 2010). The awareness of consequences can have an indirect impact on the intention of university teachers to participate in organized scientific research through three paths: behavioral attitude, personal norms, and ascribed responsibility (Wang et al., 2023). Previous studies have shown that awareness of consequences, ascribed responsibility, and personal norms have become important antecedents in social psychology for explaining and predicting individual altruistic behavior. In fact, in the specific scenario of organized scientific research in modern industrial colleges, when young teachers realize that the transformation of their research methods will positively impact the school’s development, their intention to participate may increase accordingly. Based on this, this study proposes the following hypothesis:

*H7:* Awareness of consequences positively impacts the attribution of responsibility for young teachers participating in organized scientific research.

*H8*: Awareness of consequences positively impacts young teachers' participation in organized research and personal norms.

*H9*: Ascribed responsibility positively impacts young teachers' participation in organized scientific research and personal norms.

*H10*: Personal norms have a positive impact on the intention of young teachers to participate in organized research.

### The variable relationship between planned behavior theory and normative activation theory

The Theory of Planned Behavior focuses on analyzing the antecedents of individual decision-making to maximize benefits from the perspective of self-interest. At the same time, the Theory of Normative Activation emphasizes the potential altruistic tendencies in individual behavior (Kim et al., 2018). In order to more comprehensively reflect the influencing factors of individual behavioral decisions, some scholars integrate the two theories to improve theoretical models’ predictive and explanatory power on individual behavior. For example, the subjective norms of consumers can have a significant positive impact on personal norms of their recycling behavior (Park and Ha, 2014). The residents’ awareness of the consequences of participating in environmental governance can have a significant positive impact on their behavioral attitudes (Wang and Zhang, 2017). The participation of young teachers in organized scientific research in modern industrial colleges is motivated by self-interest and altruistic tendencies that contribute to the joint development and progress of research groups. Therefore, their decision-making behavior is not entirely driven by one aspect of self-interest or altruism. Based on this, this study proposes the following hypothesis:

*H11*: Subjective norms positively impact the personal norms of young teachers participating in organized scientific research.

*H12*: Awareness of consequences positively impacts young teachers' attitudes toward participating in organized scientific research behavior.

**Figure 1:**
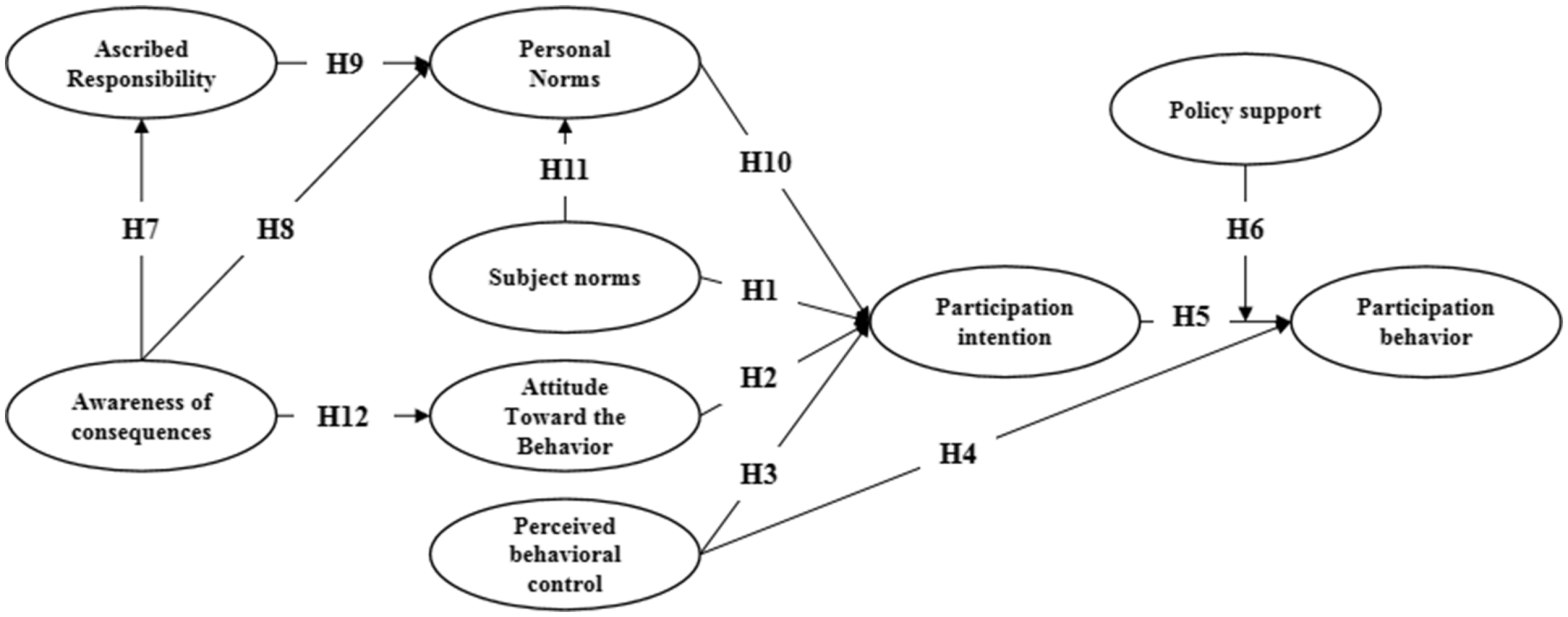
Theoretical hypothesis model of young teachers’ participation in organized scientific research behavior in Modern Industry Colleges.

Based on the above theoretical analysis and research hypothesis, this study constructs a theoretical hypothesis model for young teachers’ participation in organized scientific research behavior in modern industrial colleges and the relationship between various variables, as shown in Figure 1.

## Research design

### Scale and questionnaire design

The theoretical hypothesis model in this study covers nine variables, all of which are mature scales from existing domestic and foreign research. Semantic transformation and adaptation are based on small-scale pre-surveys and expert opinions. Subjective norms mainly drew on Ajzen (2002)'s research. We designed three measurement items from the dimensions of departmental encouragement, support from significant others, and group behavior influence (For example, young teachers around me began to try to participate in organized scientific research); The behavioral attitude mainly drew on the research of Wei and Fan (2021), we designed three measurement items from the dimensions of expanding academic resources, improving research efficiency, and promoting achievement output (For example, organized scientific research can help me acquire knowledge and resources in the required disciplines); Perceived behavioral control mainly drew on the research of Zeng (2019), we designed three measurement items from the dimensions of participation time, channels, and self-efficacy (For example, I have sufficient time to support my participation in organized scientific research); The intention to participate mainly drew on the research of Zhang et al. (2020), we designed three measurement items from the dimensions of time, energy, and recommending others (For example, I am willing to recommend organized research methods to other young teachers); Participation behavior mainly draws on the research of Wang et al. (2020), we designed three measurement items from the dimensions of past participation plans, current participation, and future deep participation (For example, I am currently involved in organized scientific research); The policy support mainly drew on the research of Song and Zhang (2023), we designed three measurement items from the dimensions of national encouragement, social appeal, and school support (For example, schools support young teachers to participate in organized scientific research); Personal norms mainly drew on Joanes (2019) research, we designed three measurement items from the dimensions of sense of obligation, moral principles, and values (For example, I believe I have an obligation to contribute to organized scientific research at the school); The attribution of responsibility mainly drew on Han’s (2014) research, which designed three measurement items from the dimensions of sense of responsibility, sense of mission, and negative impact (For example, I believe that young teachers should take some responsibility for promoting organized scientific research); Awareness of consequence mainly draws on the research of Jin (2020), we designed three measurement items from the dimensions of individual benefits, organizational benefits, and overall social benefits For example, I believe that the participation of young teachers in organized scientific research can improve overall research efficiency.

### Data and samples

The Yangtze River Delta region is one of the most active, open, and innovative regions in China’s economic development. It is also one of the regions with the most prominent industrial agglomeration effect in China. The construction and development of its modern industrial colleges have a certain representativeness nationwide. The data collection for this study relies on “Wenjuanxing” online research platform, inviting young teachers under the age of 45 from seventeen “National First Batch Modern Industry Colleges” in the Yangtze River Delta region of China to participate in filling out the questionnaire, and all participating young teachers independently complete the questionnaire options. A total of 314 questionnaire data were received. After excluding the invalid questionnaires, the remaining valid questionnaires were 309, with an effective rate of 98.14%. The sample size recommended by SEM should be ≥200 cases. The questionnaire was distributed in October 2023 using the Likert 5-point scale, representing “strongly disagree, strongly disagree, generally disagree, agree, and strongly agree” from 1 to 5 (see Table 1).

**Table 1:** Sample descriptive statistics (*N* = 309).

Category	Option	Frequency	%
Gender	Male	156	50.49%
	Female	153	49.51%
Academic qualifications	Doctor	241	77.99%
	Master or below	68	22.01%
Professional titles	Senior	107	34.63%
	Intermediate title	202	65.37%
subject	Liberal arts	126	40.78%
	Science and Engineering	183	59.22%

## Data analysis and results

### Common method bias test

To test whether the data results are affected by common method bias, this article uses Harman’s single-factor test and single factor confirmatory factor analysis for testing (Podsakoff et al., 2003). Among them, Harman’s single-factor test showed that the percentage of variance explained by the first factor before rotation was less than 50%, (Podsakoff et al., 2012) and without specifying the number of extraction factors, a total of 9 factors with eigenvalues greater than 1 were extracted; The indicators of the single factor model do not meet the reference standards (x^2^/df = 4.642, PNFI = 0.724, PCFI = 0.751, IFI = 0861, TLI = 0.840, CFI = 0.860, RMSEA = 0.109, RMR = 0.260), indicating poor fit. This suggests that there is no significant common method bias in the measurement data collected in this study.

### Reliability and validity

The reliability test uses Cronbach’s *α* and combined reliability (CR). According to Straub et al. (2004), if the variable Cronbach’s α ≥ 0.7, CR ≥ 0.7, it indicates a high level of trust in the questionnaire. Through SPSS 23.0 questionnaire analysis, it found that Cronbach’s α of each variable was Between 0.786 and 0.931, and the CR was between 0.815 and 0.931, both higher than the recommended threshold of 0.7, indicating high consistency in the measurement results of the questionnaire and high reliability (see Table 2).

**Table 2:** Reliability and convergence validity testing.

Latent variable	Observed variable	Standard loading	Cronbach’s	CR	AVE
Subjective norms (SN)	SN1	0.814	0.786	0.815	0.597
	SN2	0.834			
	SN3	0.657			
Attitude Toward the Behavior (ATB)	ATB1	0.916	0.927	0.931	0.817
	ATB2	0.937			
	ATB3	0.857			
Perceived behavioral control (PBC)	PBC1	0.736	0.848	0.842	0.640
	PBC2	0.850			
	PBC3	0.810			
Participation intention (PI)	PI1	0.838	0.857	0.860	0.670
	PI2	0.771			
	PI3	0.845			
Awareness of consequences (AC)	AC1	0.899	0.931	0.931	0.819
	AC2	0.916			
	AC3	0.900			
Ascribed Responsibility (AR)	AR1	0.802	0.840	0.848	0.651
	AR2	0.762			
	AR3	0.853			
Personal Norms (PN)	PN1	0.860	0.901	0.903	0.755
	PN2	0.857			
	PN3	0.890			
Policy support (PS)	PS1	0.876	0.871	0.876	0.703
	PS2	0.895			
	PS3	0.735			
Participation behavior (PB)	PB1	0.793	0.868	0.866	0.683
	PB2	0.785			
	PB3	0.897			

Validity testing usually includes convergent validity testing and discriminant validity testing. According to Fornell and Larcker (1981), if the standard loading is ≥0.5, the average variance extraction value (AVE) is ≥0.5, and the square root of AVE is greater than the Pearson Correlation Coefficient, it indicates that the validity of the questionnaire is high. Through AMOS23.0 questionnaire analysis, it found that the Standard loading of each measurement item was between 0.657 and 0.937 (*p* < 0.001), and the AVE was between 0.597 and 0.819, both higher than the recommended threshold of 0.5, indicating good convergent validity of the questionnaire. The square root value of AVE is more significant than the diagonal Pearson correlation coefficient, indicating good discriminant validity among the variables (see Tables 2, 3).

**Table 3:** Discriminant validity test.

Latent variable	Mean	S.D	1	2	3	4	5	6	7	8	9
1. SN	4.159	0.931	0.773								
2. ATB	4.337	0.919	0.712	0.904							
3. PBC	3.405	1.136	0.599	0572	0.800						
4. PI	4.083	0.953	0.741	0.763	0.684	0.819					
5. AC	4.449	0.777	0.660	0.723	0.433	0.780	0.905				
6. AR	3.927	1.035	0.596	0.655	0.594	0.769	0.757	0.807			
7. PN	3.941	1.027	0.600	0.673	0.610	0.813	0.677	0.916	0.869		
8. PS	4.250	0.845	0.552	0.606	0441	0.174	0.700	0.763	0.767	0.838	
9. PB	3.811	1.083	0.617	0.600	0.696	0.836	0.587	0.672	0.753	0.646	0.826

### Model fitness test

Model fit is the fitting degree between observed data and theoretical hypothesis models. The higher the fitting degree is, the stronger the usability of the model is, and the greater the practical significance of parameter estimation is (Rong, 2010). We used the maximum likelihood method to test the model’s fitness, and the results showed that x2/df = 2.930, PNFI = 0.715, PCFI = 0.742, IFI = 0.924, TLI = 0.905, CFI = 0.924, RMSEA = 0.079, RMR = 0.053. Both absolute and relative fit indexes met the requirements, indicating good fitness of the model (see Table 4).

**Table 4:** Adaptability test.

Adaptability index	Evaluation criteria	Actual value	Evaluate results
X^2^/df	<3.0	2.930	Good
CFI	>0.9	0.924	Good
TLI	>0.9	0.905	Good
RMSEA	<0.08	0.079	Good
PNFI	>0.5	0.715	Good
PCFI	>0.5	0.742	Good
IFI	>0.9	0.924	Good
RMR	<0.05	0.053	Acceptable

### Moderating effect test

**Figure 2:**
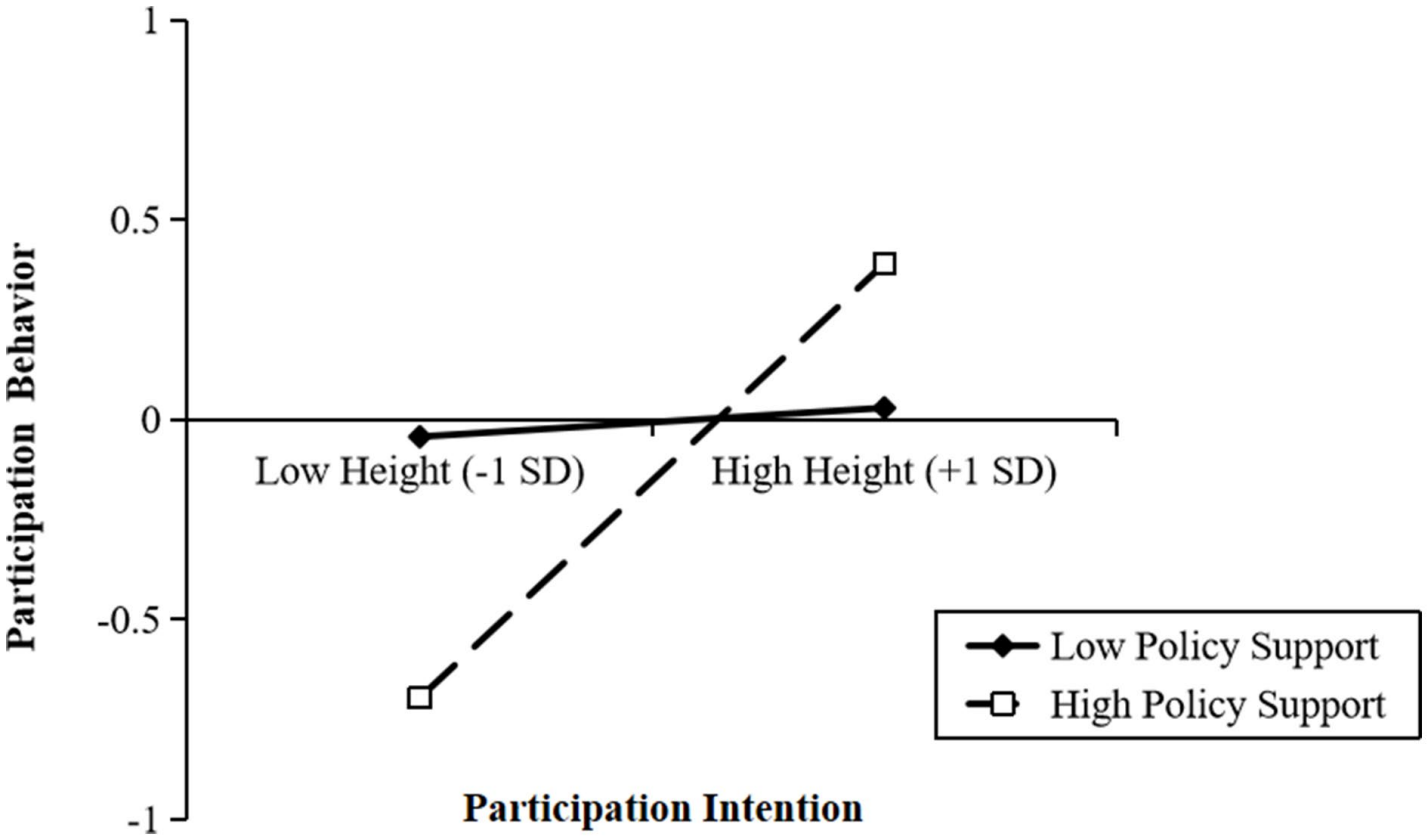
Graph of moderation effect and interaction effect.

The moderating effect reflects how the moderating variable affects the relationship between the independent and dependent variables. The moderating effect test usually uses the interaction effect test method, which means that a significant relationship between the interaction term and the dependent variable indicates a moderating effect. According to the suggestion of Wen et al. (2022), we centered the two variables of participation intention and policy support and cross-multiply them to obtain the interaction variable. The test results show that policy support significantly moderates young teachers’ participation intention and behavior (see Table 5; Figure 2).

**Table 5:** Moderating effect test.

Adjustment variable path	Estimate	S.E	C.R	*p*
PI→PB	0.839	0.092	9.079	***
PS → PB	0.146	0.085	1.722	0.085
PI*PS → PB	0.199	0.067	2.964	0.003*

*p < 0.05, ***p < 0.001, n.s. not significant.

### Path analysis and hypothesis verification

**Figure 3:**
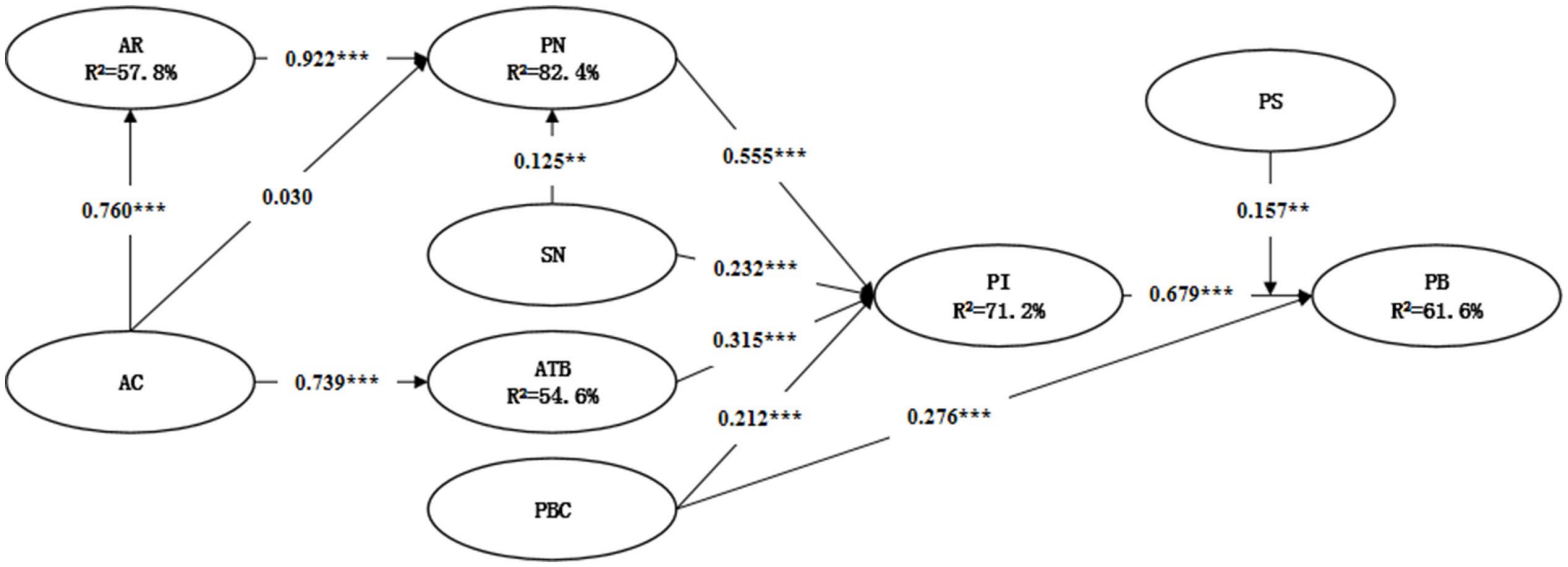
Analysis of standardized path coefficients. *p < 0.05, **p < 0.01, ***p < 0.00, n.s. not significant.

We use Amos 23.0 to perform a confirmatory analysis of the research hypothesis. The results show that subjective norms, behavioral attitudes, and perceived behavioral control significantly positively impact the young teachers’ participation intention, thus supporting H1, H2, and H3. Among them, the behavioral attitude has the most significant impact (*β* = 0.315, *p* < 0.001), followed by subjective norms (*β* = 0.232, *p* < 0.001), and finally, perceived behavioral control (*β* = 0.212, *p* < 0.001). Perceived behavioral control and participation intention significantly impact young teachers’ participation behavior, thus supporting H4 and H5. Among them, the influence of participation intention is the greatest (*β* = 0.679, *p* < 0.001), followed by perceived behavioral control (*β* = 0.276, *p* < 0.001). Policy support has a positive mediating effect on the intention and behavior of young teachers to participate (*β* = 0.157, *p* < 0.01), thus supporting H6. Ascribed responsibility and subjective norms have a significant positive impact on the personal norms of young teachers, thus supporting H9 and H11. Among them, the effect of ascribed responsibility is the greatest (*β* = 0.922, *p* < 0.001), followed by subjective norms (*β* = 0.125, *p* < 0.01). Awareness of consequences significantly impacts young teachers’ ascribed responsibility and behavioral attitude, thus supporting H7 and H12. The awareness of consequences has no significant effect on the personal norms of young teachers, thus rejecting H8. In addition, the explanatory power of the research model for the attribution of organized scientific research responsibility among young teachers is 57.8%, the explanatory power for personal norms is 82.4%, the explanatory power for behavioral attitudes is 54.6%, the explanatory power for participation intention is 71.2%. The explanatory power for participation behavior is 61.6% (see Table 6; Figure 3).

**Table 6:** Hypothesis test results.

Hypothesis path	Path coefficient	S.E.	T	*p*	Results
H1:Subject norms →Participation intention	0.232	0.046	4.590	***	Supported
H2:Attitude Toward the Behavior →Participation intention	0.315	0.044	5.793	***	Supported
H3:Perceived behavioral control →Participation intention	0.212	0.029	4.388	***	Supported
H4:Perceived behavioral control →Participation behavior	0.276	0.037	4.977	***	Supported
H5:Participation intention →Participation intention	0.679	0.044	5.793	***	Supported
H6:Policy support* Participation intention→ Participation behavior	0.157	0.067	2.964	0.003**	Supported
H7:Awareness of consequences →Ascribed Responsibility	0.760	0.059	12.902	***	Supported
H8:Awareness of consequences →Personal Norms	0.030	0.089	0.409	0.683	Unsupported
H9:Ascribed Responsibility →Personal Norms	0.922	0.110	10.216	***	Supported
H10:Personal Norms →Participation intention	0.555	0.042	9.018	***	Supported
H11:Subject Norms →Personal Norms	0.125	0.054	3.092	0.002**	Supported
H12:Awareness of consequences →Attitude Toward the Behavior	0.739	0.056	13.765	***	Supported

*p < 0.05, ***p < 0.001, n.s. not significant.

### Mediation effect test

The mediating effect reflects the indirect impact and degree of the independent variable on the dependent variable through the mediating variable. Using the Bootstrap sampling method for testing, we set up a random sampling of 5,000 times with a confidence interval of 95% (Wen et al., 2022). The test results show that the direct effect of awareness of consequence on personal norms is not significant, with an indirect effect value of 0.700 and a confidence interval of [0.555, 0.905], indicating that ascribed responsibility has a complete mediating effect on awareness of consequence; The direct effect value of subjective norms on participation intention is 0.232 (*p* < 0.001), the indirect effect value is 0.069, and the confidence interval is [0.013, 0.131], indicating that personal norms have a partial mediating effect on subjective norms, with the mediating effect accounting for 22.85%; The direct effect value of perceived behavioral control on participating behavior is 0.276 (*p* < 0.001), the indirect effect value is 0.144, and the confidence interval is [0.063, 0.242], indicating that participation intention has a partial mediating effect on perceived behavioral control, with the mediating effect accounting for 34.29%; Ascribed responsibility does not have a direct impact on intention to participate, with an indirect effect value of 0.512 and a confidence interval of [0.392, 0.657], indicating that personal norms have a complete mediating effect on ascribed responsibility; Awareness of consequence does not have a direct impact on intention, with an indirect effect value of 0.605 and a confidence interval of [0.503, 0.695]. The effect value of the path “Awareness of consequence - ascribed responsibility - personal norms – participation intention” is 0.318, with a confidence interval of [0.275, 0.456], and the mediating effect accounts for 52.56%; The effect value of the path “awareness of consequence - behavior attitude - participation intention” is 0.287, with a confidence interval of [0.194, 0.387], and the mediating effect accounts for 47.44% (see Table 7).

**Table 7:** Results of mediating effect analysis.

Mediating path	Effect value	S.E	LLCL	ULCL	*p*	Evaluate results
AC → AR → PN	0.700	0.089	0.555	0.905	***	Full mediation
SN → PN → PI	0.069	0.046	0.013	0.131	0.018*	Partial mediation
PBC → PI→PB	0.144	0.037	0.063	0.242	0.001**	Partial mediation
AR → PN → PI	0.512	0.067	0.392	0.657	***	Full mediation
AC → AR → PN → PI	0.318	0.046	0.275	0.456	***	Partial mediation
AC → ATB → PI	0.287	0.049	0.194	0.387	***	Partial mediation

*p < 0.05l, **p < 0.01, ***p < 0.00, n.s. not significant.

### Multiple-group analysis

Multi-group analysis aims to evaluate the applicability and stability of the same theoretical model across different groups. It conducts multi-group analysis by four statistical variables: gender, education level, discipline, and professional title as moderating variables. Research has found that awareness of consequence significantly impacts ascribed responsibility, ascribed responsibility on personal norms, awareness of consequence on behavior attitude, personal norms on participation intention, behavior attitude on participation intention, and participation intention on participation behavior in various young teacher groups. The impact of consequence awareness on personal norms in different young teacher groups is insignificant. It is consistent with the overall sample, indicating no significant group difference in the above functional relationship regarding gender, education level, discipline, and professional title.

Regarding gender, subjective norms have no significant impact on the personal norms of young male teachers but have a substantial impact on young female teachers. It may be related to the psychological resilience of young male and female teachers. Generally speaking, young male teachers have a relatively strong ability to withstand pressure and relieve stress. In contrast, young female teachers are more sensitive and delicate in their emotions due to the back-and-forth transition between family and professional roles. Especially under specific external pressures, they may be more inclined to choose to accept group values and moral standards. In terms of education, perceived behavioral control has no significant impact on the participation behavior of young doctoral teachers. Still, it significantly affects young teachers with master’s degrees or below. It may be related to young teachers’ scientific literacy and research ability. Generally speaking, young doctoral teachers who have received complete and comprehensive research training have relatively strong research abilities and a higher sense of self-efficacy in overcoming research obstacles and difficulties. Young teachers with a master’s degree or below, due to their relative lack of research experience, prefer to use organized research to enhance their abilities. Regarding disciplinary categories, subjective norms have no significant impact on the personal norms and participation intention of young teachers in science and engineering but substantially affect the group of young teachers in liberal arts. It may be related to young teachers’ disciplinary thinking patterns and characteristics. Generally speaking, scientific research in science and engineering has a strong dependence, and its research activities exhibit a highly specialized and collaborative “flow production.” In contrast, in liberal arts, scientific research is a highly individualized “craftsman-style production.” They prefer to break away from traditional “individual combat” and “academic isolation” through organized research. Regarding professional titles, subjective norms significantly impact the personal norms of young teachers with senior professional titles but have no significant impact on intermediate and lower-level young teachers. It may be related to the driving force behind young teachers conducting academic research. Generally speaking, the research motivation of young teachers with low professional titles mainly stems from their relentless pursuit of professional title promotion rather than external moral obligations and social responsibilities. Comparatively, young teachers with senior professional titles bear greater social responsibility in their research field, and they prefer to form research teams through organized research to improve research innovation efficiency and social influence. In addition, perceived behavioral control has no significant impact on the intention of senior professional title young teachers to participate. Still, it significantly impacts intermediate and below young teachers, which may be related to the academic resource endowment of young teachers. Generally speaking, young teachers with senior professional titles have relatively abundant academic resources, and most have even formed their research teams. However, young teachers with lower professional titles have limited academic resources and limited resource channels. Therefore, they hope to improve their academic level and produce results through organized scientific research (see Table 8).

**Table 8:** Multi-group analysis of young teachers participating in organized scientific research behavior.

Path	Gender		Academic qualifications		Subject		Professional title	
	Male	Female	Doctor	Master or below	Science and Engineering	Liberal arts	Senior	Intermediate or below
AC → AR	0.751***	0.807***	0.746***	0.766***	0.715***	0.760***	0.704***	0.788***
AR → PN	0.968***	0.971***	0.931***	0.886***	0.873***	0.910***	0.838***	0.995***
SN → PN	0.097	0.145*	0.030	0.232***	−0.055	0.252***	0.136*	0.090
AC → ATB	0.809***	0.692***	0.846***	0.694***	0.791***	0.744***	0.798***	0.728***
AC → PN	0.040	0.136	0.044	0.085	0.122	0.085	0.032	0.085
PN → PI	0.519***	0.541***	0.415***	0.633***	0.628***	0.422***	0.473***	0.565***
SN → PI	0.243***	0.167*	0.185**	0.296***	0.035	0.330***	0.247***	0.217***
ATB → PI	0.356***	0.414***	0.478***	0.238***	0.275**	0.408***	0.461***	0.299***
PBC → PI	0.218**	0.139*	0.170*	0.209***	0.271***	0.209***	0.027	0.275***
PBC → PB	0.179*	0.413***	0.136	0.284***	0.364***	0.228**	0.279**	0.230***
PI→PB	0.763***	0.581***	0.793***	0.662***	0.622***	0.688***	0.787***	0.665***

*p < 0.05, **p < 0.01, ***p < 0.001, n.s. not significant.

## Conclusion and enlightenment

This study is based on the theory of planned behavior and normative activation. It constructs an integrated model for the participation of young teachers in organized scientific research behavior in modern industrial colleges. Based on 309 questionnaire data, we use the Amos structural equation to analyze the research hypothesis, mediation effects, and group differences. The following conclusions are as follows: firstly, personal norms, subjective norms, behavioral attitudes, and perceived behavioral control have a significant positive impact on the intention of young teachers to participate in organized scientific research, and personal norms have the most significant impact. It differs to some extent from the conclusion drawn by Wang et al. (2023) that the behavioral attitude has the greatest impact on the university teachers’ participation intention in organized scientific research, and it may be related to the increasingly fierce phenomenon of “involution” among young teachers; Secondly, the awareness of consequences, ascribed responsibility, and subjective norms have an indirect effect on the intention of young teachers to participate in organized scientific research. The indirect effect of consequence awareness is generated through “awareness of consequence-ascribed responsibility-personal norms-participation intention” and “awareness of consequence-behavior attitude-participation intention.” The indirect effect of ascribed responsibility and subjective norms is generated through personal norms. It is consistent with the research conclusion of Wang (2022). Thirdly, participation intention and perceived behavioral control have a significant positive impact on the participation of young teachers in organized scientific research, and the influence of participation intention is the greatest. It is consistent with the research conclusion of Qin et al. (2020). Fourthly, policy support has a positive mediating effect on the intention and behavior of young teachers to participate in organized scientific research. It is consistent with the research conclusion of Zhang and Song (2024). Fifth, there are group differences in the subjective attribution, personal norms, perceived behavioral control, participation intention, and participation behavior of young teachers in organized scientific research. It is consistent with the research conclusion of Li and Chen (2023). Based on the above conclusion, we can give inspiration to the following four points:

(1) Improving the internal interest coordination mechanism of scientific research organizations to enhance the sense of scientific research achievement of young teachers

From the perspective of self-interest, subjective norms, behavioral attitudes, and perceived behavioral control have a significant positive impact on the intention of young teachers to participate in organized scientific research. In other words, in the real scenario of scientific research activities in modern industrial colleges, expanding scientific research resources, improving scientific research efficiency, obtaining team support, and organizational recognition are important internal driving forces for young teachers to participate in organized scientific research, which is consistent with the research findings of Liu and Li (2022). Generally speaking, most modern industrial colleges implement a research team leader system for organized research, which is to form an academic community with professors or authoritative experts as the core of the team (Wang et al., 2019). The advantage of this organizational form is that academic resources can be autonomously allocated within the team. The disadvantage is that the team leader has absolute control over the allocation of academic resources, which can easily lead to an imbalance in the allocation of academic resources within the team. For example, research platform leaders, primary project hosts, and the first signatories of various awards are often highly concentrated under the team leader’s name. Under the existing scientific research evaluation mechanism, this imbalance or even conflict of interests can harm the enthusiasm of other team members to some extent, especially for newly hired young teachers, who may even form the illusion that participating in organized scientific research is equivalent to working for professors. Therefore, in promoting organized scientific research, modern industrial colleges should establish and improve a scientific and reasonable internal team interest coordination mechanism to enhance the young teachers’ sense of achievement in organized scientific research. For example, based on the existing team leader system, we should explore establishing a benefit distribution mechanism guided by scientific research participation, investment, contribution, etc., to enhance the enthusiasm of young teachers to participate in organized scientific research.

(2) Restructuring the organized scientific research evaluation model of modern industrial colleges to enhance the teamwork awareness of young teachers

From the perspective of altruism, personal norms, sense of responsibility, and consequences have a significant direct or indirect positive impact on the intention of young teachers to participate in organized scientific research. In other words, young teachers view promoting scientific and technological progress or team development through organized scientific research as a social responsibility and moral obligation, consistent with the research findings of Guo and Yang (2021). Zhao (1998) believes that with the increasing organizational level of cutting-edge technological innovation, scientific research individuals can only become a part of the scientific cause and impact the history of science by effectively integrating themselves into the entire scientific research community. The purpose of organized scientific research conducted by modern industrial colleges is to gather scientific research forces through team collaboration, target cutting-edge technology and significant national needs, and carry out original and leading technological breakthroughs. As far as the current research evaluation model in domestic universities is concerned, due to the excessive emphasis on indicators such as first author, first inventor, and first unit in professional title evaluation and performance evaluation, the results can easily lead to “cooperation without cooperation” and “non-cooperation without cooperation” among research team members. Teachers with slight potential are willing to work alone (Zhang and Liu, 2023), detrimental to scientific research cooperation among teachers. It also needs to be more conducive to consolidating scientific research efforts. Therefore, in promoting organized scientific research, modern industrial colleges should break the long-term and highly rigid “individualistic” evaluation model, reconstruct a multidimensional evaluation system that emphasizes individual and team evaluation, and further enhance the teamwork awareness of young teachers.

(3) Improving the support system for organized scientific research policies to stimulate the self-efficacy of young teachers

From the perspective of behavior occurrence, policy support has a significant positive moderating effect on the intention and behavior of young teachers to participate in organized scientific research. In other words, the more the policy support system tends to be improved, the more helpful it is for young teachers to transform organized scientific research intentions into practical actions, which is consistent with the research conclusion of Wang et al. (2019). Generally speaking, transforming teacher research methods or reshaping research habits is a process from “external driving” to “internal self-discipline,” which requires specific external incentives. Due to the lack of research platforms and the unclear “head goose” effect of scientific research, scientific research organizations’ overall “adsorption power” is not strong. Teachers’ research activities are mainly loose: “individual combat” and “do-it-alone,” especially for some newly hired young teachers who often wander outside the research team, making it difficult to form a research aggregation force. Therefore, in promoting organized scientific research, modern industrial colleges need to strengthen policy guidance and support from the top-level design, such as building interdisciplinary cooperation platforms, setting up special support funds, establishing a youth teacher mentor system, etc., to guide young teachers to integrate into existing research teams or form new teams actively. In addition, perceived behavioral control has a significant positive impact on the participation of young teachers in organized scientific research behavior. It also requires modern industrial colleges to consider the “up and down connection” and “left and right connection” of policies in formulating organized policies, further stimulating the self-efficacy of young teachers and promoting organized scientific research to organized innovation.

(4) Paying attention to the group differences among young teachers and creating a research environment that is tolerant of failure.

From the perspective of group differences, there are significant group differences in young teachers’ participation in organized scientific research in five aspects: subjective norms, personal norms, perceived behavioral control, participation intention, and participation behavior. Specifically, female teachers, liberal arts teachers, teachers with master’s or below degrees, and teachers with intermediate or below professional titles have a more vital intention and active behavior to participate in organized scientific research. The reason for this is that under the current digital laws such as “scientific research competition,” “academic GDP,” and “scientific research KPI” (Zhang and Liu, 2023), the tendency of schools to take actions such as “up-or-transfer,” “up-or-out, “and “supporting the strong but not the weak” have caused varying degrees of psychological anxiety for such teachers. They both expect to use organized research to enhance their personal research output and fear being overlooked or rejected due to insufficient abilities. Therefore, in promoting organized scientific research, modern industrial colleges should fully consider the differences among young people with different disciplines, educational backgrounds, and professional titles, create a research environment that tolerates failure, and establish a sound corresponding fault-tolerant mechanism.

## Limitations and future research

Considering the limitations of many objective factors, this study used young teachers from the seventeen “National First Batch of Modern Industry Colleges” in the Yangtze River Delta region of China as the research samples. However, these types of industry colleges were not classified, and the sample size was small, which may affect the applicability of the research conclusions. In the future, it is necessary to expand the sample size further based on the classification of industrial colleges to enhance the widespread application of research conclusions.

## Data Availability

The original contributions presented in the study are included in the article/supplementary material, further inquiries can be directed to the corresponding author.
